# Evidence-informed policy making at country level: lessons learned from the South African Tuberculosis Think Tank

**DOI:** 10.5588/ijtld.17.0485

**Published:** 2018-06-01

**Authors:** R. G. White, S. Charalambous, V. Cardenas, P. Hippner, T. Sumner, F. Bozzani, D. Mudzengi, R. M. G. J. Houben, D. Collier, M. E. Kimerling, A. Vassall, Y. Pillay, G. Churchyard

**Affiliations:** * TB Modelling Group, Centre for the Mathematical Modelling of Infectious Diseases, and Department of Infectious Disease Epidemiology, London, UK; † TB Centre, London School of Hygiene & Tropical Medicine, London, UK; ‡ Aurum Institute, Johannesburg, South Africa; § School of Public Health, University of Witwatersrand, Johannesburg, South Africa; ¶ Advancing Treatment and Care for TB/HIV, South African Medical Research Council, Johannesburg, South Africa; # Aurum Institute, Washington DC, USA; ** WhiteOx, Bristol, UK; †† KNCV Tuberculosis Foundation, The Hague, The Netherlands; ‡‡ South African National TB Control Programme, Pretoria, South Africa

**Keywords:** analysis, modelling, policy, decision making, evaluation

## Abstract

**BACKGROUND:**

National Tuberculosis Programmes (NTPs) require specialist input to support the development of policy and practice informed by evidence, typically against tight deadlines.

**OBJECTIVE:**

To describe lessons learned from establishing a dedicated tuberculosis (TB) think tank to advise the South African NTP on TB policy.

**INTERVENTION AND EVALUATION METHODS:**

A national TB think tank was established to advise the NTP in support of evidence-informed policy. Support was provided for activities, including meetings, modelling and regular telephone calls, with a wider network of unpaid expert advisers under an executive committee and working groups. Intervention evaluation used desktop analysis of documentary evidence, interviews and direct observation.

**RESULTS:**

The TB Think Tank evolved over time to acquire three key roles: an ‘institution’, a ‘policy dialogue forum’ and an ‘interface’. Although enthusiasm was high, motivating participation among the NTP and external experts proved challenging. Motivation of working groups was most successful when aligned to a specific need for NTP decision making. Despite challenges, the TB Think Tank contributed to South Africa's first ever TB and human immunodeficiency virus (HIV) investment case, and the decision to create South Africa's first ever ring-fenced grant for TB. The TB Think Tank also assisted the NTP in formulating strategy to accelerate progress towards reaching World Health Organization targets.

**DISCUSSION:**

With partners, the TB Think Tank achieved major successes in supporting evidence-informed decision making, and garnered increased funding for TB in South Africa. Identifying ways to increase the involvement of NTP staff and other experts, and keeping the scope of the Think Tank well defined, could facilitate greater impact. Think tank initiatives could be replicated in other settings to support evidence-informed policy making.

## Abstract (FR)

**CONTEXTE:**

Les Programmes Nationaux Tuberculose (PNT) requièrent la participation de spécialistes pour soutenir le développement d'une politique et de pratiques basées sur des preuves, généralement dans un délai très court.

**OBJECTIF:**

Décrire les leçons apprises lors de la mise en place d'un groupe de réflexion tuberculose (TB) dédié pour conseiller la politique du PNT sud-africain en matière de politique relative à la TB.

**METHODES D'INTERVENTION ET D'EVALUATION:**

Un groupe de réflexion national TB a été mis en place pour conseiller le PNT en vue d'une politique basée sur des preuves. Un soutien a été fourni pour diverses activités : réunions, modélisation et appels téléphoniques réguliers à un large réseau de conseillers bénévoles dans le cadre d'un comité exécutif et de groupes de travail. L'évaluation de l'intervention a utilisé une analyse de preuves documentaires, d'entretiens et d'observation directe.

**RÉSULTATS:**

Le groupe de réflexion TB a évolué dans le temps vers trois rôles clés, une institution, un forum de dialogue politique et une interface. Bien que l'enthousiasme ait été élevé, motiver la participation du PNT et d'experts externes s'est avérée être un défi. La motivation des groupes de travail a eu plus de succès quand elle a été alignée à un besoin spécifique en matière de prise de décision du PNT. En dépit des défis, le groupe de réflexion TB a contribué au tout premier dossier d'investissement TB-VIH (virus de l'immunodéficience humaine) d'Afrique du Sud et à la décision de créer la toute première dotation allouée à la TB en Afrique du Sud. Le groupe de réflexion TB a également assisté le PNT dans la formulation de la stratégie visant à accélérer les progrès vers les objectifs de l'Organisation Mondiale de la Santé.

**CONCLUSION:**

Le groupe de réflexion TB, avec ses partenaires, a abouti à des succès majeurs en soutenant la prise de décision basée sur des preuves et a obtenu une augmentation du financement de la TB en Afrique du Sud. Identifier des moyens d'augmenter l'implication du personnel du PNT et d'autres experts et maintenir le champ d'application du groupe de réflexion bien défini, pourrait favoriser un meilleur impact. Les initiatives de groupe de réflexion pourraient être répliquées dans d'autres contextes afin de soutenir la prise de décision basée sur des preuves.

## Abstract (ES)

**MARCO DE REFERENCIA:**

Los Programas Nacionales contra la Tuberculosis (PNT) precisan la contribución de especialistas con el fin de respaldar la formulación de políticas y prácticas documentadas en pruebas fidedignas, sobre todo cuando se deben cumplir plazos estrictos.

**OBJETIVO:**

Describir las lecciones aprendidas con la instauración de un grupo de reflexión específico sobre tuberculosis (TB), cuyo objeto era prestar asesoramiento al PNT de Suráfrica en materia de políticas contra la TB.

**INTERVENCIÓN Y MÉTODOS DE EVALUACIÓN:**

Se instauró un grupo de reflexión sobre TB con miras a asesorar al PNT y respaldar las políticas basadas en pruebas fidedignas. Este grupo de reflexión, estructurado en un comité ejecutivo y grupos de trabajo, prestó apoyo a diversas actividades como las reuniones, la modelización y las llamadas periódicas a una red más amplia de asesores expertos no remunerados. La intervención se evaluó mediante el análisis de fuentes secundarias como las pruebas documentales y las entrevistas, además de la observación directa.

**RESULTADOS:**

Con el transcurso del tiempo, las funciones del grupo de reflexión evolucionaron hacia tres misiones principales, a saber: una institución, un foro de diálogo sobre políticas y una interfase. Aunque existía un gran entusiasmo, fue difícil motivar la participación de miembros del PNT y otros expertos externos. La motivación de los grupos de trabajo fue más eficaz cuando se armonizaba con una necesidad específica del PNT para la toma de decisiones. Pese a las dificultades, el grupo de reflexión sobre TB contribuyó al primer caso de inversión conjunta contra la TB y el VIH (virus de la inmunodeficiencia humana) en la historia de Suráfrica y a la decisión de crear la primera subvención reservada a la TB en el país. El grupo de reflexión ayudó también al PNT a formular una estrategia encaminada a acelerar el progreso hacia las metas de la Organización Mundial de la Salud.

**CONCLUSIÓN:**

El grupo de reflexión sobre TB, con sus asociados, alcanzó grandes logros al respaldar la toma de decisiones documentadas en pruebas fidedignas y consiguió un aumento del financiamiento contra la TB en Suráfrica. La determinación de mecanismos que fomenten la participación del personal del PNT y otros expertos y la definición clara de las competencias del grupo de reflexión, podría favorecer una mayor repercusión de su acción. Es posible reproducir las iniciativas del grupo de reflexión en otros entornos con el propósito de respaldar la formulación de políticas basadas en datos fidedignos.

TUBERCULOSIS (TB) presents a major health burden in South Africa.^1^ In response to this problem, and with considerable recent political support,^2–4^ South Africa's National TB Programme (NTP) has become an early adopter of innovation.^5^ However, resources are limited and the NTP requires specialist input to support the development of policy and practice, typically against tight deadlines.

International bodies such as the World Health Organization (WHO) provide global TB guidelines and periodic country epidemiological reviews, but these global bodies cannot provide the rapid, ‘bespoke’ advice that the South African and other NTPs often require.^6^

As such, a dedicated TB think tank was conceived by the NTP to fill the gap, drawing on existing national expertise and research capacity, and international networks. The Think Tank was tasked with anticipating and responding to policy makers' requests for evaluation of evidence and quantitative analysis, and with improving TB data utilisation.

The need for rapid, bespoke advice is not unique to South Africa or TB. Advisory bodies and agencies have been created in many countries worldwide to fill this advice gap in low/middle-7–9 and high-income counties.^10^ In South Africa itself, an HIV Think Tank also exists, tasked with ‘. . . providing a central place for all stakeholders [under Department of Health], to review epidemiological, routine monitoring and economic evidence related to the HIV epidemic, identify priority gaps, and establish consensus on appropriate next steps, including research projects and pilots of new programs and policies’.^11^ However, very little has been written about the structure and effectiveness of health policy think tanks.^8^,^12–14^ Reviewing this literature, Bennett et al. concluded that a small number of factors were key to the success of health policy think tanks: production of timely, relevant, credible, trustworthy and actionable evidence, and close relationships with policy makers.^12^ In their in-depth analysis of six health policy think tanks in Bangladesh, Ghana, India, South Africa, Uganda and Viet Nam, Bennett et al. also concluded that a supportive policy environment, some degree of independence from the government, and strong links to policy makers were critical for effective policy engagement. A study on the formation of a think tank-like institution in Indonesia identified challenges that included longer-term financial support, a limited number of scientific publications and difficulties in documenting the impact of the think tank on programmatic performance.^9^

To contribute to this critical, but limited, literature, the aim of the present study was to describe the lessons learned from establishing a dedicated TB Think Tank to advise the South African NTP on TB policy.

## INTERVENTION AND EVALUATION METHODS

### Intervention mission and structure

The mission of the TB Think Tank was to advise the NTP on anti-tuberculosis treatment and prevention policy and programmatic implementation to achieve the National Strategic Plan (NSP)/World Health Assembly targets for TB, with a special focus on innovations. The internal structure of the TB Think Tank evolved over time, and its current organisational structure is shown in the Figure. The Think Tank, which was created to include an Executive Committee and three expert working groups, was co-chaired by the Deputy Director General for Health Strategy and the head of a South African research institute. The three expert working groups were each chaired by two or more co-chairs, including an NTP staff member and another individual (or two) from domestic expert organisations:

1) Working group 1: Modernising a national response to TB aligned to the Post-2015 Global TB Programme Strategy, including:
  - Know your epidemic (systematic data analysis)
  - Define your interventions (e.g., access to care and active case finding)
  - Plan your response (including modelling and economics)
2) Working group 2: Implementation and delivery, including:
  - Information and communication technologies, monitoring and evaluation, and surveillance
  - Forecasting and budgeting
  - Monitoring implementation of new policy
  - Human resources planning and training
3) Working group 3: Research on diagnostics, drugs and vaccines

**Figure:**
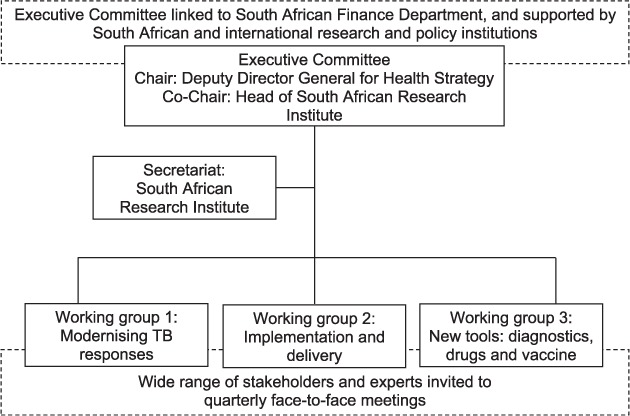
Current organisational structure of the South Africa TB Think Tank. SA=South Africa. TB = tuberculosis.

The South African Government Health and Finance Departments formed the core of the Think Tank, supported by South African and international research institutions, including the Department of Science and Technology, the South Africa Medical Research Council (MRC, Cape Town, South Africa), the London School of Hygiene & Tropical Medicine (London, UK), technical support agencies, funders and the WHO Global TB Programme and UNAIDS (Geneva, Switzerland).

### Intervention objectives

The wider project supporting the Think Tank had five main objectives: 1) formalising the TB Think Tank; 2) creating and applying epidemiological and economic modelling tools to identify cost-effective and affordable strategies to assess, and reach, NSP goals; 3) promoting the use of the quantitative evidence generated by these tools to inform TB prevention and care policy and practice in South Africa; 4) building capacity and sustainable systems to ensure that the tools can be used within the country to inform TB prevention and care policy and practice; and 5) assessing the success of the project in disseminating findings and, if successful, identifying funding to support systems beyond the end of the project.

### Intervention activities and resources

The TB Think Tank was designed to consider policy and implementation questions requiring evidence to inform policy by carrying out the following activities: 1) collating, reviewing, synthesising and evaluating evidence; 2) requesting evidence and, if necessary, commissioning research; 3) brainstorming innovations and making recommendations to the NTP for policy and implementation in the form of policy briefs; 4) assisting the NTP in developing operational guidelines; and 5) advising the NTP on key implementation activities in support of budget discussions with the National Treasury and as part of investment case development for new donor grants. The Think Tank was set up with financial support from the Bill and Melinda Gates Foundation (BMGF). This funded quarterly face-to-face Think Tank meetings, the dedicated epidemiological modelling and economic staff (~3.5 people) and convening of regular telephone calls with a wider network of unpaid expert advisers in the Executive Committee and the three area working groups. Table 1 shows the Think Tank activities for the example of childhood screening for TB.

**Table 1:**
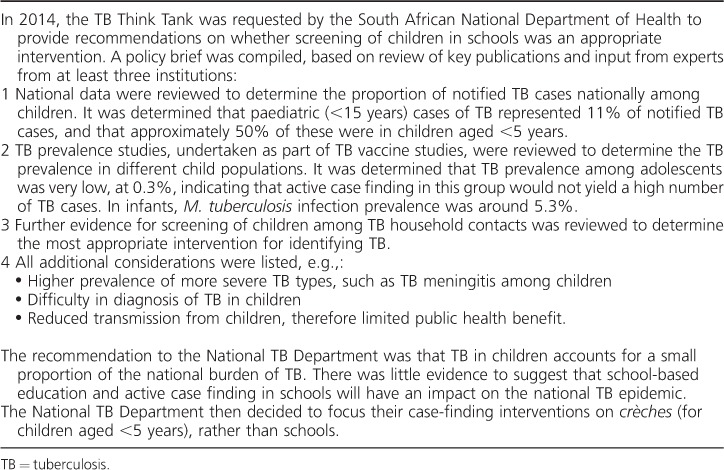
Illustrative example of Think Tank processes: childhood screening for TB

### Intervention operating modes

The Think Tank was established flexibly to operate in two modes: 1) to respond to specific, usually time-limited, requests from the NTP; and 2) to serve as ‘thought drivers’ for large national strategy development processes such as the new 5-year NTP Strategy.

## EVALUTION METHODS

This BMGF project was evaluated by an independent external evaluator (DC) based on desktop analysis of documentary evidence, interviews and direct observation. The main audience was the project sponsors (the funder and the NTP) and other key stakeholders. The evaluation was also designed to provide the project team with regular constructive feedback to help improve the likelihood of a successful outcome. It recognised that the project's evolutionary nature and organisational change themes were often best supported though an ‘appreciative inquiry’ approach, focusing more on identifying and building on what was working well and less on correcting problems or deviations from the detail of the original proposal. The funder was amenable to this approach.

The evaluator's synthesis of the evidence and his conclusions and recommendations were published in his March 2017 Final Evaluation Report, which drew on:

1) Baseline interviews during June–September 2014 with project team members and external stakeholders and a review of project documents (Interim Report 1).
2) Project team interviews and a review of progress reports during July–December 2015 (Interim Report 2).
3) Participant interviews and observation of the December 2015, April 2016 and August 2016 TB Think Tank and NTP workshops (Interim Reports 2 and 4).
4) An analysis of think tank models and evolving TB Think Tank roles (Interim Report 5).
5) Final evaluation interviews November–December 2016 with project sponsors, international and national stakeholders, project team members and an audit of project management documentation and procedures.

All interviews were non-attributable. The project team was invited to comment on factual accuracy before publication, but the evaluator retained complete discretion over its inclusion. Recommendations included in the present study were based on findings from the evaluator's external evaluation.

## RESULTS AND LESSONS LEARNED

### Main achievements

With its partners, the Think Tank achieved several major successes over the first 2 years (Table 2). Soon after the Think Tank was launched, impact modelling, carried out by modellers supporting the Think Tank, was used to help define the scope of a South African MRC/UK MRC funding call on operational research, with a budget of 70 million South African rand (ZAR).

**Table 2:**
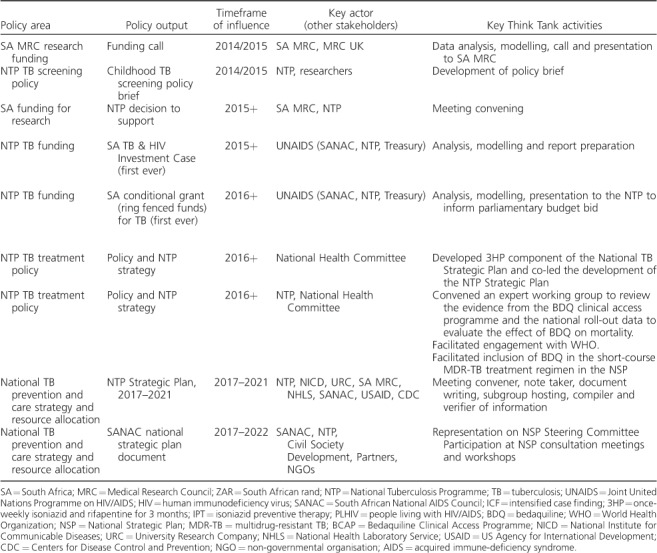
Examples of likely policy influence of the TB Think Tank, 2014–2016

**Table 2:**
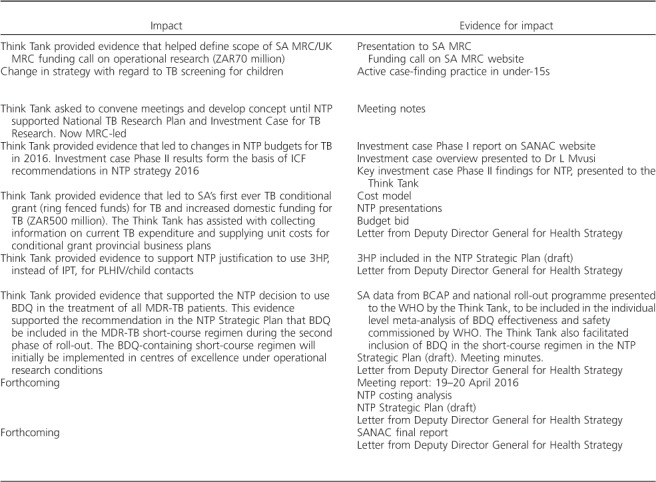
(*continued*)

In 2015, the Think Tank supported the establishment of the National TB Research Plan and Investment Case for TB Research by convening meetings and developing the initial concept note. Also in 2015, the Think Tank provided evidence supporting the creation of South Africa's first ever joint TB and HIV Investment Case. This led to changes in NTP budgets for TB in 2016, and formed the basis of intensified case finding recommendations in the new NTP strategy.

In 2016, the Think Tank provided evidence that led to South Africa's first ever TB conditional grant (ring-fenced funds) for TB, and increased domestic funding for TB by ZAR 500 million. Later that year, the Think Tank provided evidence that informed the NTP decision to use ‘3HP’ preventive therapy for people living with HIV and child contacts, instead of ‘IPT’. Also in 2016, the Think Tank provided evidence that supported the NTP decision to use bedaquiline (BDQ) in the treatment of all multidrug-resistant TB patients. This evidence supported the recommendation in the NTP Strategic Plan that BDQ be included in the short-course MDR-TB regimen during the second phase of roll-out.^15^ The BDQ-containing short-course regimen will initially be implemented in centres of excellence under operational research conditions. The Think Tank also took a leading role in supporting the NTP development of South Africa's new NTP Strategic Plan 2017–2021. The Think Tank convened meetings on the Plan, compiled and verified information from experts and created a first draft of the Plan for NTP finalisation. The Plan has yet to be formally released, but it will shape the TB response in South Africa for the next 5 years. The Think Tank also has representation on the South African National AIDS council (SANAC) Steering Committee, which brings together government, civil society and the private sector to create a collective response to HIV, TB and sexually transmitted infections. The Think Tank provided TB evidence for the new SANAC NSP to ensure alignment with the NTP TB Department's Strategic Plan.

### Main challenges and enablers

Although initial enthusiasm was high, motivating active and sustained participation among NTP staff and external experts proved challenging. From its initiation, it was evident that the broad active participation of NTP staff was essential for the success of the Think Tank. However, there were real constraints in terms of the amount of time that individual NTP staff could dedicate to the Think Tank in the absence of a mandate from the NTP leadership and/or provision of relief from other duties. The same was true of external experts, who were time-constrained and funded largely through grants; the ability to provide a substantial amount of unfunded time was therefore limited. There was also a perception that the Think Tank agenda was overly driven by key partners. This then became the challenge for the Secretariat: to facilitate open and transparent Think Tank calls and meetings that included robust and sustained NTP and external expert participation.

Within 1 year of the formation of the Think Tank, it became evident to the Secretariat that the required level of stakeholder input would be hard to achieve. Due to scant participation at its programmed monthly teleconferences, the Secretariat abandoned the monthly format in favour of quarterly face-to-face meetings. Also evident was the need to ‘flesh out’ robust agendas, provoke vigorous discussion and provide a platform for active decision making. There was a general perception that these quarterly Think Tank meetings were to be different from typical TB conferences where researchers are asked to present their latest research work and findings to a general (TB) audience, with little input required from the audience. Rather, NTP staff and external experts demanded meeting agendas at which their targeted input was required in the form of yes/no votes on, for example, the implementation of Strategy A vs. Strategy B.

The Secretariat thus worked with the Think Tank co-chairs to create meeting agendas that identified the 2–3 highest priority items for the NTP, e.g., whether ultraviolet lamps should be used for infection control and the value of serological tests for the diagnosis of Mycobacterium tuberculosis infection and TB disease. Once chosen, the Secretariat reached out to experts, collated the evidence required for presentation to the Think Tank and organised the meeting with the goal of provoking vigorous debate, but also consensus building and decision-making.

By reducing the frequency of the Think Tank meetings to a quarterly format, and by focusing the agenda on 2–3 priority items requiring concrete input from its members, the Secretariat could mobilise vigorous participation from both the NTP staff and external experts, and channel this participation to result in concrete suggested paths of action for NTP strategy.

The roles and activities of the Think Tank greatly expanded over time in response to the needs of the NTP: first, as a science-based ‘institution’ providing robust and independent evidence for policy formation, usually in response to an NTP request for rapid evidence. One example was providing evidence on the potential use of 3HP therapy for people living with HIV and child contacts (Table 2). A second example was a ‘policy dialogue forum’ facilitating wider engagement between the NTP and stakeholders on TB policy, e.g., facilitating the development of the NTP NSP (Table 2). A third example was an ‘interface’ between the NTP and the modelling/economics community, where resource constraints limited the NTP's ability to act as a critical consumer of research output.

In addition, other enablers included strong political support from the South African President and Minister of Health for improving TB prevention and care, core funding for convening activities and analytical work and, over time, improving communication channels between the NTP, modellers, economists and other experts.

### Summary of independent evaluation, future directions and sustainability

The Think Tank was set up to be both demand- and supply-driven, i.e., responding to requests originating in the NTP, but also investigating issues originated by its working parties. However, over time, it evolved towards the demand end of the spectrum. In response, its remit and processes have developed to find a balance that matches national need.

Key elements now need to be addressed to ensure the future sustainability and utility of the Think Tank. Perhaps most importantly, decision makers and funders should consider balancing the benefits of continuing to use the Think Tank in its three roles (‘institution’, ‘policy dialogue forum’ and ‘interface’) against the risk that its resources will be spread too thinly and it will fail to fully deliver any of these roles.

### Institutional role

In its ‘institutional’ role, the Think Tank placed more emphasis on responding to government needs rather than proactively promoting the Think Tank members' analysis of key priorities. Under this model, the Think Tank operated more as an integral part of the NTP policy development process; it was sponsored by the NTP, but carried out work at arm's length from ministers. Although there are as yet only a few completed examples (e.g., development of policy briefs), the Think Tank has shown it can be an independent body, supporting decision making by advising on issues put to it by the Government. Improvements can be made: feedback from the NTP on decisions eventually taken often did not reach the contributing experts; it is therefore essential in future to maintain engagement and to help members keep advice relevant. Some saw this reactive institutional role as less valuable, but we believe that this role may in fact be more critical over the long term, as it has the benefit of being aligned to the NTP's expressed needs, and has built the NTP's trust and belief in the utility of the Think Tank. We suggest that the working groups could still originate issues under an institutional model. In addition, to further strengthen the institutional aspects of the Think Tank, it could be structured in a way similar to other organisations that give arm's length advice, such as the National Institute of Clinical Excellence in the UK, so that it does not require NTP ‘ownership’, which may compromise its independence. Finally, for the institution to function more efficiently, we propose improved linkages between the TB Think Tank and other think tanks, particularly the HIV Think Tank.^11^

### Role as a policy dialogue convener

The NTP encouraged the Think Tank from the start to take on a significant ‘policy dialogue convener’ role across initiatives and funders. In future, this could continue to be a major secondary benefit, but only if it is clear which organisation is responsible for which function or programme. This complementary policy dialogue role has proved very valuable to the NTP and National TB Strategic Plan stakeholders, including a wide variety of national institutions and non-governmental organisations, and deserves to be explicitly supported. However, if it is not covered explicitly in future funding agreements, we warn that the Think Tank may end up doing the job again anyway, with consequences for both the quality of the dialogue and the other work of the Think Tank. In addition, to further strengthen this role of the Think Tank, the NTP needs to be enabled to take increased ownership for the Think Tank by taking on (or having seconded) a dedicated member of staff responsible for the Think Tank. We also recommend that external experts be better enabled to contribute to the Think Tank, perhaps by funding their time directly, or with citeable acknowledgment of their contribution to NTP decision making. We recommend that the Think Tank be better integrated into the existing longer-term planning cycles in South Africa, as has occurred in case of the SANAC 2017–2022 NSP. There is also a need to change any perception that the Think Tank agenda is overly driven by key partners, perhaps by diversifying Think Tank membership. Finally, the Think Tank needs sustained funding, with a plan to transition to NTP funding over time.

### Interface role

In its third role, the Think Tank has also provided value as an interface between the NTP and modelling initiatives, and has provided resources where constraints limited the NTP's ability to act as an ‘intelligent customer’ of analytical work. It helped the NTP formulate its requirements and interpreted/packaged modelling outputs for NTP use. However, TB modelling skills are extremely rare in South Africa. We therefore recommend short-term funding specifically to support TB modelling until other South African institutions can maintain TB modelling expertise, so that the Think Tank can draw on these skills when required for specific (usually short-term) Think Tank tasks. A grant commissioning management ability could also be included in the Think Tank funding renewal to facilitate more engagement with a wider range of experts.

### Comparison with other Think Tank initiatives

There are similarities between the challenges and enablers identified in previous research on health policy think tanks^9^,^12^ and those in our study. The South African TB Think Tank was fortunate to have a supportive policy environment, some degree of independence from the Government, and strong links to policy makers, which facilitated effective policy engagement. Despite challenges, the South African TB Think Tank also tended to provide timely, relevant, credible, trustworthy and actionable evidence to the NTP that further strengthened the relationship between the Think Tank and the NTP over time. The South African TB Think Tank was also fortunate in that it has so far only suffered temporary shortfalls in financial support, unlike the longer-term shortfalls experienced by think tanks in Bangladesh, India, Uganda and Indonesia.^9^,^12^

Unlike the Indonesian think tank,^9^ the evaluation of the South African TB Think Tank focused on the strategic and process level pre-conditions for impact on TB control, rather than practice level impacts. This was a deliberate decision, taken at the time of the creation of the Think Tank, because it was thought impact on practice was unlikely over the ~2 years of the initial funding. However, given that alleviating suffering from TB (via changes in practice) is the ultimate goal of the Think Tank, perhaps the impact of the Think Tank on practice, as well as policy, could be evaluated in the current funding cycle.

We believe that the likely utility of this Think Tank, and the contribution of other health policy think tanks,^9^,^12^ justify their wider application to support evidence-informed TB decision-making. Some key characteristics for effective engagement and practical delivery have now been identified, and many aspects of the South African Think Tank model could be replicated in other settings.

## CONCLUSION

The South African TB Think Tank, with its partners, achieved major successes in supporting evidence-informed decision-making, and garnered increased funding for TB management in South Africa. Identifying ways to increase the involvement of NTP staff and other experts, and keeping the scope of the Think Tank well defined, could lead to greater impact. Think tank initiatives could be replicated in other settings to support evidence-informed policy making.
